# The Effect of Novel Research Activities on Long-term Survival of Temporarily Captive Steller Sea Lions (*Eumetopias jubatus*)

**DOI:** 10.1371/journal.pone.0141948

**Published:** 2015-11-18

**Authors:** Courtney Shuert, Markus Horning, Jo-Ann Mellish

**Affiliations:** 1 School of Fisheries and Ocean Sciences, University of Alaska Fairbanks, Fairbanks, AK, 99775, United States of America; 2 Alaska SeaLife Center, 301 Railway Ave, Seward, AK, 99664, United States of America; 3 Department of Fisheries and Wildlife, Oregon State University, Newport, OR, 97365, United States of America,a; 4 North Pacific Research Board, Anchorage, AK, 99501, United States of America; Sonoma State University, UNITED STATES

## Abstract

Two novel research approaches were developed to facilitate controlled access to, and long-term monitoring of, juvenile Steller sea lions for periods longer than typically afforded by traditional fieldwork. The Transient Juvenile Steller sea lion Project at the Alaska SeaLife Center facilitated nutritional, physiological, and behavioral studies on the platform of temporary captivity. Temporarily captive sea lions (TJs, *n* = 35) were studied, and were intraperitoneally implanted with Life History Transmitters (LHX tags) to determine causes of mortality post-release. Our goal was to evaluate the potential for long-term impacts of temporary captivity and telemetry implants on the survival of study individuals. A simple open-population Cormack-Jolly-Seber mark-recapture model was built in program MARK, incorporating resightings of uniquely branded study individuals gathered by several contributing institutions. *A priori* models were developed to weigh the evidence of effects of experimental treatment on survival with covariates of sex, age, capture age, cohort, and age class. We compared survival of experimental treatment to a control group of *n* = 27 free-ranging animals (FRs) that were sampled during capture events and immediately released. Sex has previously been show to differentially affect juvenile survival in Steller sea lions. Therefore, sex was included in all models to account for unbalanced sex ratios within the experimental group. Considerable support was identified for the effects of sex, accounting for over 71% of total weight for all *a priori* models with delta AICc <5, and over 91% of model weight after removal of pretending variables. Overall, most support was found for the most parsimonious model based on sex and excluding experimental treatment. Models including experimental treatment were not supported after *post-hoc* considerations of model selection criteria. However, given the limited sample size, alternate models including effects of experimental treatments remain possible and effects may yet become apparent in larger sample sizes.

## Introduction

The two distinct population segments (east and west) of Steller sea lions (*Eumetopias jubatus*) have been the subject of extensive study in the past few decades due to substantial decline in some portions of their range (e.g., [1,2]). As part of the intensive research effort to better understand the population dynamics of the western population segment, two novel approaches were developed in order to gain extended access to wild individuals and to determine potential causes of mortality. Temporary captivity, an approach here referred to as the Transient Juvenile Steller sea lion Project (TJ) was implemented to gain greater access to individuals, while attempting to minimize disturbance to the population at large [3]. The project has facilitated studies with nutritional, physiological, and behavioral contributions [4–8]. Long-term tracking of these individuals (Transient Juveniles, TJs) was facilitated through hot-iron brands on their left flank upon release as mandated by terms of project-specific handling authorization by the federal government. An additional cohort of catch-and-release, branded, free-ranging sea lions (*n* = 27, FRs) served as a control group, similar to those of other institutions using brand-resight methods for population monitoring (e.g., [9]). Life History Transmitters (LHX tags; [10]) were intraperitoneally implanted into *n* = 35 TJs under standard aseptic surgical procedures and gas anesthesia [11], starting in 2005 and through 2011. LHX have a projected life span of 10 or more years and generate end-of-life, post-mortem known-fate data [9,10,11,12].

The current analysis was implemented to evaluate the effect on long-term survival of these two novel approaches on endangered Steller sea lions. Many efforts have been made to study the survival and behavior of Steller sea lions utilizing external satellite and dive tags [7,13], as well as mark-resight studies of flipper tagged and hot-iron branded individuals [14–16]. However, the impact of these studies on survival is typically only documented in the short-term, with an assumption of little impact after the handling event. This is especially true of studies involving tagging of animals for resight purposes, but has not been evaluated in many species due to logistical constraints [17,18]. The analysis presented here was facilitated by a decade of shared data from multiple institutions, allowing for a survival analysis of treatment groups.

The Cormack-Jolly-Seber (CJS) model design is an open population mark-recapture model that includes survival and recapture or resight probabilities, relying on live encounters [19,20]. Encounter histories of individuals consist of simple logistic data organizing each encounter occasion as presence and absence during a resighting period. Models predicting survival within these groups are then based on covariates in various combination, both continuous and categorical [21]. Using this design template, we assessed survival between the control (FR) and experimental groups that participated in captivity and received LHX implants (TJ) with the addition of several demographic covariates.

## Methods

### Study animals

All work was carried out under National Marine Fisheries Service permits #881–1668, 881–1890, 14335. All work was approved by the Alaska SeaLife Center, Oregon State University and University of Alaska Fairbanks IACUCs. Included in this study, 62 juvenile Steller sea lions were captured via underwater lasso technique between 2005 and 2011 [3,22]. Thirty-five of these individuals (TJs) were retained for temporary captivity for research purposes up to a maximum of three months and received dual LHX implants, with only two individuals receiving a single implant at the start of the project [3,11]. Of the TJs, 27 were male and 8 were female. Twenty-seven animals were sampled, hot-iron branded and immediately released (FRs, 12 male and 15 female, [3,23]). All individuals received a unique 4-digit alphanumeric brand. Age at capture was determined through canine length as per King et al. (2007) as the approximate age in months used to back calculate to the closest mean peak pupping date, June 10^th^, to get an estimated birth date [24]. Three individuals did not have their canines measured during sampling and were aged from a standard length-to-age correlation [25]. Study animals were captured at a mean age of 1.6 ± 0.51 years as estimated by canine length or extrapolation from standard length, and binned in a 1 (14–24 months) or 2 (25–36 months) year age group.

### Resights and source agencies

Branded animal resight information was gathered from multiple source agencies operating in Alaska from May 15^th^, 2005 through August 30^th^, 2013. Contributing sources were the National Marine Mammal Laboratory (7600 Sand Point Way, Seattle, Washington 98115), Alaska Department of Fish & Game (1255 W. 8th Street, Juneau, Alaska 99811), and the Chiswell Project at the Alaska SeaLife Center (301 Railway Ave, Seward, Alaska 99664). The first two sources provided resights as conducted by yearly cruise efforts to survey haulouts and rookeries largely during the summer months in South Central and Southeastern Alaska (averaging 148 and 111 days per year, respectively, pers. comm. K. Hastings and R. Towell). The third source only focused on the Chiswell Island rookery and nearby haulouts surveyed by remote video monitoring in the central Gulf of Alaska continuously through the summer breeding season and periodically through the rest of the year (avg. 333 days per year, pers. comm. J. Maniscalco). All brand resights were compiled into a database with at minimum the location, date, brand readability, and observer confidence in the accuracy of the resight. Only those resights that could be confirmed with an accompanying photograph were used in this analysis.

### Model development

All resight data for each brand was reorganized into a simple binary code encounter history for use and input into program MARK [20]. Individual encounter histories contained nine resight intervals between the months of March and November from 2005 to 2013. Each resight year was set at a default ‘0’ for no resight events, and a ‘1’ if a resight occurred, regardless of the frequency. Models were developed *a priori* and parameterized around demographic covariates of sex, age, age at capture (14–24 or 25–36 months grouping bins), cohort group, and time. Age class was also included as a covariate with two juvenile classes of 14–24 months and 25–36 months of age as well as a single adult cohort, including animals older than 36 months, to account for differential juvenile and adult survival. Sex, age at capture, and cohort were also included separately as time-dependent factors. Models were subsequently ranked using Akaike Information Criterion model selection methods and corrected for small sample size (AICc, [20,26,27]). These covariates were used for building survival and resight probabilities through the CJS method for estimation. Resight effort was included in all p models as a covariate in order to properly scale yearly differences in institutional effort and prevent inflation of resight probabilities. Resight effort data in the form of number of resighting survey days in a given year was normalized on a scale of 1 to 10. Each model was run through MARK via the RMark package [28]. Grouping variables for all animals included the age an individual was first sampled to account for differences in ages within resighting periods in addition to sex, and cohort groups.

Our analysis included our experimental group that experienced temporary captivity and received LHX implants (TJs, *n* = 35) as well as those that were marked and released immediately following capture (FRs, *n* = 27). Demographic covariates detailed above were also included, with models doubled to have an identical set that included a model term (TJFR) to test the relative importance of our experimental group in model ranking. Best models, deemed to have considerable support in model ranking, were determined as being within 3 ΔAICc values, but were also considered equivalent and indistinguishable in their support level [29]. These highest ranked models were then used to generate comparison sets of survival and resighting probability models. Models within 5 ΔAICc were deemed to have minor support in the data and were also discussed in their implications for our study. Goodness-of-fit testing was used for global models of each grouping factor to assess the potential for model overfitting through the program U-CARE [30]. Sex and treatment had Chi-squared values that reflected non-significant P-values at an alpha value of 0.05. The derived c-hat values were all approximately equal to 1, so no adjustments were necessary for *a priori* models for overdispersion.

## Results

A Fisher’s exact test found that the sex ratio was skewed within groups by comparing the actual ratios in experimental groups to an equal sex ratio contingency table. TJs had a significantly skewed sex ratio (*p* = 0.02), and FRs did not (p = 0.81). A Fisher’s exact test also found that age at capture was significantly skewed between TJs and FRs (*p* = 0.001), but not within the TJs alone (*p* = 0.29). In all extant studies, age and sex have been shown to have the strongest and most consistent effects on the survival of juvenile pinnipeds [1,9,15,31]. Therefore, sex was included as a mandatory covariate in all models. The known effect of age on survival is diminishing with increasing age, and capture age was therefore not forced into each model.

Model ranking results and beta values for top models are included in S1 and S2 Tables, respectively. Top ranked models (≤ 3 ΔAICc) included sex (Rank 1) and the additive effect of sex and experimental group factor (Rank 2). The two top models (≤ 3 ΔAICc) shared 91% of the overall model weight. However, for all nested models the addition of the experimental group factor consistently resulted in an increase of approximately two AICc units (see S1 Table), suggesting the possibility of this being a ‘pretending variable’ [32]. Appearance of the experimental group factor was inconsistent (in models ranked 2, 4, 7 and 8), and addition of the factor altered the deviance by 0.3% or less in all cases (S1 Table). The treatment factor beta parameter estimate for the highest ranked model including the factor (2) exhibits large confidence intervals that span zero (S2 Table), and was the case for all models including the treatment. Together, these considerations support the notion that the addition of the experimental group factor does not explain any additional variance in response; the difference in AICc is simply driven by the penalty associated with the addition of a variable. This in turn leads to the *post-hoc* removal of models 2, 4, 7 and 8 from consideration [32]. Model weights were then re-computed, and led to an evidence ratio for our top model of 10.4, found in Table 1.

**Table 1:** Final model results for the apparent survival of juvenile Steller sea lions (*Eumetopias jubatus*).

Model	*k*	Delta AICc	Weight	Deviance
Phi(~Sex)p(~Sex + Age + Effort)	13	0	0.912	229.071
Phi(~Sex + Age Class)p(~Sex + Age + Effort)	15	4.687	0.088	229.038

Our experimental group parameter was ultimately removed due to its lack of fitting improvement. These models represent the most parsimonious with the most support found (≤ 5 delta AICc) in our analysis of assessing long-term survival. Models demonstrating differential survival between our control and those that were temporarily captive and received LHX implants were deemed not plausible and were removed due to their suspected inclusion of pretending variables.

**Fig 1:**
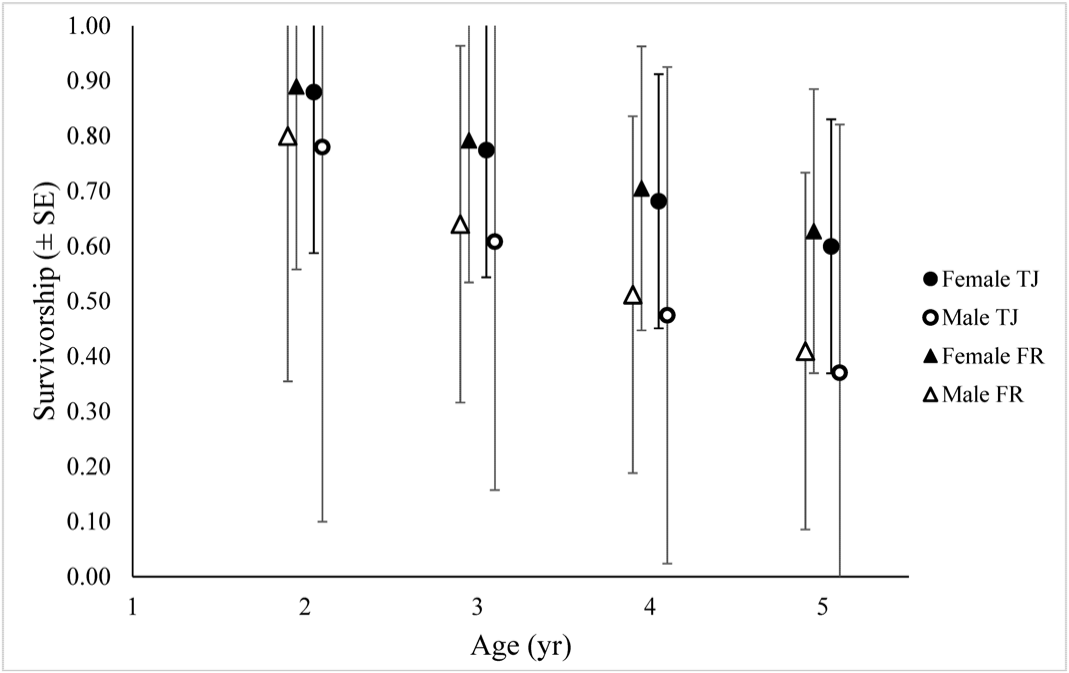
Model averaged cumulative survival for juvenile Steller sea lions (*Eumetopias jubatus*) participating in temporary captivity. Cumulative survival rates for animals aged 2 to 5 comparing survivorship between males (open) and females (closed) as well as those that were held in temporary captivity receiving LHX implants (TJ, circles) and those that were released immediately after the initial sampling event (FR, triangles). 95% confidence intervals for each term also included as capped lines.

The resulting ranking provides substantial evidence that within the models considered and our limited data set, sex has a considerable effect on survival. Males had a lower averaged survival than females (Table 2). Mean survival rates for individuals in the experimental group were slightly lower than our control group, though confidence intervals widely overlap (Table 2). Minor support (≤ 5ΔAICc) was also identified for the additive effect of age class and sex on survival, but was only weighted 8.8% of all models (Table 1). No support was ultimately identified for the effects of experimental treatment, age, cohort, or capture age, nor were any covariates made time-dependent. Cumulative survival rate from 15 mo to age 5 for the TJ group overall was calculated to be 0.43 based on generated annual age-specific survival rates (Fig 1). Model averaged resighting probabilities are presented in Table 3. All demographic data and brand resighting histories for study individuals are contained in S3 and S4 Tables, respectively.

**Table 2:** Model-averaged apparent survival rates from top Cormack-Jolly-Seber models predicting long-term survival in juvenile Steller sea lions (*Eumetopias jubatus*).

				95% Confidence	
		Estimate	Standard Error	Lower	Upper
Experimental (TJLHX)	Male	0.783	0.044	0.684	0.857
	Female	0.882	0.041	0.773	0.942
Control (FR)	Male	0.797	0.047	0.688	0.875
	Female	0.891	0.033	0.805	0.942

Survival was compared between our experimental group (TJLHX) of juvenile Steller sea lions that experienced temporary captivity as well as LHX implantation to a wild control group (FR). Apparent survival rates here are representing the averaged mean annual survival over the total resighting period from 15 months to 9 years of age. Estimated standard errors and 95% confidence intervals also included.

**Table 3:** Resighting probabilities for temporarily captive juvenile Steller sea lions (*Eumetopias jubatus*).

				95% Confidence	
	Age	Estimate	Standard Error	Lower	Upper
Male	2	0.504	0.291	0.093	0.909
	3	0.193	0.098	0.065	0.451
	4	0.553	0.127	0.311	0.772
	5	0.774	0.097	0.535	0.911
	6	0.790	0.092	0.558	0.918
	7	0.89	0.073	0.65	0.972
	8	0.81	0.096	0.553	0.936
	9	0.81	0.147	0.394	0.965
Female	2	0.695	0.225	0.221	0.948
	3	0.348	0.129	0.148	0.621
	4	0.735	0.109	0.478	0.893
	5	0.885	0.065	0.687	0.964
	6	0.894	0.061	0.705	0.967
	7	0.947	0.04	0.787	0.988
	8	0.905	0.058	0.713	0.973
	9	0.905	0.079	0.606	0.983

Model averaged resighting probabilities, standard error, and 95% confidence intervals for top predictors (sex and age) of Cormack-Jolly-Seber models predicting return rate and long-term survival in juvenile Steller sea lions (*Eumetopias jubatus*).

## Discussion

Survival rates are well understood for wild animals where handling for tagging is minimal, but the impact of more intensive sampling and temporary captivity had yet to be determined. In the current study, our analysis was used to investigate potential long-term effects of two novel research techniques utilized on juvenile Steller sea lions through evidence-based model ranking. We solely used demographic covariates of project individuals including sex, age, age class, experimental cohort, and age at capture as well as time-dependent forms of these covariates.

After the identification of a considerable sample size bias by sex in the experimental treatment group, only those models that included sex as a covariate were considered. Maniscalco [31] reported survival rates based on resights of 199 Steller sea lions branded as pups on the Chiswell Island rookery in our study region between 2005 and 2010. He reported cumulative survival rates from the age of 3 weeks through 4 years of 35.7% (+/- 8.2% S.E.), for all animals pooled. From the published results [31] we back-calculated a cumulative survival estimate of 49.8% for the ages of 15 months to 4 years, for comparative purposes. From Maniscalco’s published results, and for sex ratios equivalent to the experimental treatment group and control groups, we estimated cumulative survival rates of 43.5% and 52.3%, respectively, and of 47.4% for the combined groups. Our own estimate of 49.1% is very close to the overall value from Maniscalco, and actually above the value for a comparable sex ratio. This suggested a strong likely effect of our sex bias in the treatment group on group specific and also overall survival rates. The mean survival rate was indeed slightly higher for females in our study group (Fig 1, Table 2). This is consistent with findings in many species of pinnipeds, including Steller sea lions [9,33], grey seals (*Halichoerus grypus*, [34]), Galapagos and northern fur seals (*Arctocephalus galapagoensis* and *Callorhinus ursinus*, [35]) and others.

Resighting probability model ranking was also found to be influenced by sex and age, but not as time-specific covariates. This is consistent with other studies [31,33]. Juvenile males tend to disperse farther than females [13,14], which may explain in part why males were found to most often have a lower resighting probability than female groups (see Table 3). Several study animals were seen within the range of the eastern population post-release, adding to recent evidence that suggests that the distinction by eastern and western stock may have to be revisited [16]. Since the CJS model cannot separate out permanent emigration from mortality, it is possible that several males may have simply emigrated out of the main resight effort area, both lowering their estimated resighting probability as well as their apparent survival rates.

Mean apparent survival probability differed between the experimental treatment and control groups (Table 2), but the magnitude of this effect was much smaller than uncertainties. The top ranked survival model that initially carried 64% of model weights was also the most parsimonious model based on sex as the main predictor. Addition of the experimental treatment factor in the second ranked model that carried 26% of model weights did not result in an improved fit. Since the treatment factor beta parameter confidence estimates spanned zero we deemed the difference in AICc as most likely resulting from the numerical penalty associated with the addition of a variable to the model [32]. When such ‘pretending variables’ are removed from consideration, model weights should be re-computed [32]. The so corrected weights lead to a single model with delta AICc<5, and an evidence ratio of 10.4. Thus, the simplest model had more than ten times the support of the second model in the revised rankings, and models that include the experimental treatment are not supported by the analysis following our *post-hoc* evaluation. This judgment is supported by the finding that our overall survival estimate is slightly above a comparable estimate back-calculated from Maniscalco 2014 [31]. However, given the limited sample size, alternate models including effects of experimental treatments remain possible and effects may yet become apparent in larger sample sizes.

### Other considerations and conclusions

A power analysis in program MARK returned an effective sample size of 211 animals. While we corrected for our small sample size by utilizing AICc, which by nature penalizes models with more complex structure much more harshly than AIC [29], our sample size substantially constrained our ability to detect minute differences in survival, as evidenced by the large overlapping confidence intervals associated with group mean survival differences.

To date, we have not been able to collect evidence in support of negative effects of two novel research techniques, temporary captivity and implantation of LHX, on the survival of endangered Steller sea lions. Animals in our study appear to be exhibiting similar or even higher mean survival rates to rates reported from separate mark resight studies in the region during the same period. However, given our low power of the absence of findings of negative effects of the new research approaches of temporary captivity combined with transmitter implantation cannot be seen as a proof of the absence of any effects. A precautionary approach to the continued application of these novel research techniques could involve the selective use of only one or the other technique for specific research projects, until larger sample sizes are reached.

## Supporting Information

S1 Table Survival model selection results for juvenile Steller sea lions assessing the impact of experiencing temporary captivity and LHX implantation. Model selection results for assessing the impact of demographics covariates as well as the experimental impact of temporary captivity and LHX implants (TJFR) against a wild control group on the long-term survival of juvenile Steller sea lions (*Eumetopias jubatus*) incorporating covariates of sex, age class (A_3_), capture age (Ac), age, cohort (C), time and effort (Ef). Models were built under Cormack-Jolly-Seber assumptions with selection based on corrected Akaike Information Criterion (AICc). Results presented here include only the top 12 models for brevity. Best models with the most support were considered to be within 2 delta AICc and are highlighted in **bold**. Models within 5 delta AICc were also considered to have minor support. ^**§**^Models ultimately excluded from the final results. The TJFR factor did not improve model fit, nor did it change the overall deviance in comparative models and was therefore removed from model selection.
(DOCX)

S2 Table Beta values for temporary captivity and LHX implant survival models in juvenile Steller sea lions. Top model beta parameters for survival probability (Φ, Phi) and resight probability (p) for models assessing the effect of LHX-1 implants and temporary captivity on juvenile Steller sea lions (*Eumetopias jubatus*) as determined by being within 2 delta AICc model rankings of a Cormack-Jolly-Seber mark-recapture format.
(DOCX)

S3 Table Demographics, brand and implantation data used for evaluating long-term survival in juvenile Steller sea lions. Demographic, brand, cohort, and implantation summary data used to evaluate long-term survival between LHX-1 implanted, temporarily captive (TJ) Steller sea lions and a control of free-ranging juveniles (FR) including sex (Male/Female), age at capture (CapAge), cohort (IndocDate), and group (TJ/FR). This data was used to create covariates for a priori Cormack-Jolly-Seber models assessing survival through AICc model selection methods.
(DOCX)

S4 Table Individual brand resighting summary for evaluating survival in juvenile Steller sea lions. Summary of resighting events on an annual basis for both temporarily captive, implanted (prefix TJ-) and free-ranging (prefix FR-) juvenile Steller sea lions. Each individual brand resight history was reduced to a binary encounter history for input into Program MARK for survival analysis. The first instance of a resight or null place holder (-) indicates the year that the animal was marked and released with the resighting events summed in the left column (‘Resights’). Only those resights that included a photograph to confirm a positive identification were included in this analysis. Resights of these study individuals were gathered from various contributing institutions including the National Marine Mammal Laboratory, the Alaska Department of Fish & Game, and the Alaska SeaLife Center.
(DOCX)
