# Supplementary material for: The Effect of Novel Research Activities on Long-term Survival of Temporarily Captive Steller Sea Lions (Eumetopias jubatus)
Source: PLoS One. 2015 Nov 18;10(11):e0141948. doi: 10.1371/journal.pone.0141948 (PMC4651490; doi:10.1371/journal.pone.0141948)
Supplement: S1 Table — Model selection results for assessing the impact of demographics covariates as well as the experimental impact of temporary captivity and LHX implants (TJFR) against a wild control group on the long-term survival of juvenile Steller sea lions (Eumetopias jubatus) incorporating covariates of sex, age class (A3), capture age (Ac), age, cohort (C), time and effort (Ef). Models were built under Cormack-Jolly-Seber assumptions with selection based on corrected Akaike Information Criterion (AICc). Results presented here include only the top 12 models for brevity. Best models with the most support were considered to be within 2 delta AICc and are highlighted in bold. Models within 5 delta AICc were also considered to have minor support. §Models ultimately excluded from the final results. The TJFR factor did not improve model fit, nor did it change the overall deviance in comparative models and was therefore removed from model selection. (DOCX) [file pone.0141948.s001.docx]

**S1 Table. Survival model selection results for juvenile Steller sea lions assessing the impact of experiencing temporary captivity and LHX implantation.**

| (a) Model | *k* | AICc | Delta AICc | Model Weight | Deviance |
| --- | --- | --- | --- | --- | --- |
| **Phi(~Sex)p(~Sex + Age + Ef)** | **13** | **340.2285** | **0** | **0.6487** | **229.0712** |
| **Phi(~Sex + TJFR)p(~Sex + Age + Ef)^§^** | **14** | **342.0327** | **1.8042** | **0.2632** | **228.5293** |
| Phi(~Sex + A_3_)p(~Sex + Age + Ef) | 15 | 344.9158 | 4.6872 | 0.0622 | 229.0381 |
| Phi(~Sex + A_3_ + TJFR)p(~Sex + Age + Ef) **^§^** | 16 | 346.7068 | 6.4783 | 0.0254 | 228.4263 |
| Phi(~Sex + time)p(~Sex + Age + Ef) | 20 | 356.3873 | 16.1587 | 0.0002 | 228.1987 |
| Phi(~Sex + Age)p(~Sex + Age + Ef) | 21 | 358.5139 | 18.2854 | 0 | 227.7714 |
| Phi(~Sex + Age + TJFR)p(~Sex + Age + Ef) **^§^** | 22 | 360.4047 | 20.1762 | 0 | 227.0763 |
| Phi(~Sex + Age + Ac + TJFR)p(~Sex + Age + Ef) **^§^** | 23 | 362.6737 | 22.4451 | 0 | 226.7269 |
| Phi(~Sex)p(~Sex *time + Age + Ef) | 27 | 363.2328 | 23.0043 | 0 | 216.4746 |
| Phi(~Sex + Age + C)p(~Sex + Age + Ef) | 29 | 363.9084 | 23.6798 | 0 | 211.5324 |
| Phi(~Sex + TJFR)p(~Sex*time + Age + Ef) **^§^** | 28 | 365.3069 | 25.0784 | 0 | 215.7580 |
| Phi(~Sex + Age + C + TJFR)p(~Sex + Age + Ef) | 30 | 366.7034 | 26.4749 | 0 | 211.4632 |
| … | … | … | … | … | … |
| Phi(~Sex + C + time)p(~Sex*time + Age + Ef) | 81 | 476.1359 | 59.9339 | 0 | 245.3019 |

Model selection results for assessing the impact of demographics covariates as well as the experimental impact of temporary captivity and LHX implants (TJFR) against a wild control group on the long-term survival of juvenile Steller sea lions (*Eumetopias jubatus*) incorporating covariates of sex, age class (A_3_), capture age (Ac), age, cohort (C), time and effort (Ef). Models were built under Cormack-Jolly-Seber assumptions with selection based on corrected Akaike Information Criterion (AICc). Results presented here include only the top 12 models for brevity. Best models with the most support were considered to be within 2 delta AICc and are highlighted in **bold**. Models within 5 delta AICc were also considered to have minor support.

**^§^**Models ultimately excluded from the final results. The TJFR factor did not improve model fit, nor did it change the overall deviance in comparative models and was therefore removed from model selection.
