# Supplementary material for: The Effect of Novel Research Activities on Long-term Survival of Temporarily Captive Steller Sea Lions (Eumetopias jubatus)
Source: PLoS One. 2015 Nov 18;10(11):e0141948. doi: 10.1371/journal.pone.0141948 (PMC4651490; doi:10.1371/journal.pone.0141948)
Supplement: S2 Table — Top model beta parameters for survival probability (Φ, Phi) and resight probability (p) for models assessing the effect of LHX-1 implants and temporary captivity on juvenile Steller sea lions (Eumetopias jubatus) as determined by being within 2 delta AICc model rankings of a Cormack-Jolly-Seber mark-recapture format. (DOCX) [file pone.0141948.s002.docx]

**S2 Table. Beta values for temporary captivity and LHX implant survival models in juvenile Steller sea lions.**

|  |  |  |  |  | 95% Confidence Interval | |
| --- | --- | --- | --- | --- | --- | --- |
| Model | | Parameter | Beta | Standard Error | Lower | Upper |
| Phi(~ Sex) | p(~Sex + Age + Effort) | Phi(Intercept) | 2.0860 | 0.3410 | 1.4176 | 2.7544 |
|  |  | Phi(Sex) | -0.7685 | 0.4129 | -1.5778 | 0.0408 |
|  |  | p(Intercept) | 2.7316 | 13.662 | -24.046 | 29.509 |
|  |  | p(Sex) | -0.8037 | 0.4909 | -1.7660 | 0.1586 |
|  |  | p(Age 3) | -1.0727 | 2.9757 | -6.9053 | 4.7598 |
|  |  | p(Age 4) | 0.1916 | 1.1944 | -2.1495 | 2.5328 |
|  |  | p(Age 5) | 1.2128 | 1.2326 | -1.2031 | 3.6287 |
|  |  | p(Age 6) | 1.6849 | 2.9422 | -4.0818 | 7.4517 |
|  |  | p(Age 7) | 2.0654 | 1.3300 | -0.5413 | 4.6722 |
|  |  | p(Age 8) | 1.4191 | 1.2580 | -1.0467 | 3.8849 |
|  |  | p(Age 9) | 1.4088 | 1.3987 | -1.3326 | 4.1502 |
|  |  | p(Age 10) | 15.5462 | 3925.8 | -7679.1 | 7710.2 |
|  |  | p(Effort) | -0.3805 | 2.7242 | -5.7201 | 4.9589 |
| Phi(~Sex + TJFR)* | p(~Sex + Age + Effort) | Phi(Intercept) | 1.8598 | 0.4486 | 0.9803 | 2.7392 |
|  |  | Phi(Sex) | -0.6489 | 0.4411 | -1.5135 | 0.2156 |
|  |  | Phi(TJFR) | 0.3037 | 0.4125 | -0.5047 | 1.1123 |
|  |  | p(Intercept) | 2.7428 | 13.696 | -24.102 | 29.588 |
|  |  | p(Sex) | -0.8070 | 0.4920 | -1.7714 | 0.1573 |
|  |  | p(Age 3) | -1.0777 | 2.9829 | -6.9243 | 4.7689 |
|  |  | p(Age 4) | 0.1862 | 1.1970 | -2.1599 | 2.5325 |
|  |  | p(Age 5) | 1.2050 | 1.2348 | -1.2153 | 3.6254 |
|  |  | p(Age 6) | 1.6854 | 2.9496 | -4.0958 | 7.4667 |
|  |  | p(Age 7) | 2.0671 | 1.3327 | -0.5450 | 4.6793 |
|  |  | p(Age 8) | 1.4438 | 1.2612 | -1.0280 | 3.9158 |
|  |  | p(Age 9) | 1.4860 | 1.4309 | -1.3186 | 4.2907 |
|  |  | p(Age 10) | 15.408 | 3515.1 | -6874.3 | 6905.1 |
|  |  | p(Effort) | -0.3817 | 2.7310 | -5.7346 | 4.9711 |

Top model beta parameters for survival probability (Φ, Phi) and resight probability (p) for models assessing the effect of LHX-1 implants and temporary captivity on juvenile Steller sea lions (*Eumetopias jubatus*) as determined by being within 2 delta AICc model rankings of a Cormack-Jolly-Seber mark-recapture format.

*Model ultimately excluded from model development.
