# Supplementary material for: The Effect of Novel Research Activities on Long-term Survival of Temporarily Captive Steller Sea Lions (Eumetopias jubatus)
Source: PLoS One. 2015 Nov 18;10(11):e0141948. doi: 10.1371/journal.pone.0141948 (PMC4651490; doi:10.1371/journal.pone.0141948)
Supplement: S3 Table — Demographic, brand, cohort, and implantation summary data used to evaluate long-term survival between LHX-1 implanted, temporarily captive (TJ) Steller sea lions and a control of free-ranging juveniles (FR) including sex (Male/Female), age at capture (CapAge), cohort (IndocDate), and group (TJ/FR). This data was used to create covariates for a priori Cormack-Jolly-Seber models assessing survival through AICc model selection methods. (DOCX) [file pone.0141948.s003.docx]

**S3 Table. Demographics, brand and implantation data used for evaluating long-term survival in juvenile Steller sea lions.**

| **Individual** | **Sex** | **CapAge** | **Brand** | **Implanted** | **TJFR** | **Cohort** | **IndocDate** |
| --- | --- | --- | --- | --- | --- | --- | --- |
| FR035 | F | 2 | =905 | - | FR | 2005 Sep | 03-Sep-05 |
| FR036 | F | 2 | =904 | - | FR | 2005 Sep | 03-Sep-05 |
| FR037 | F | 1 | =906 | - | FR | 2005 Sep | 05-Sep-05 |
| TJ022 | F | 2 | =908 | LHX-1 | TJ | 2005 Sep | 07-Sep-05 |
| TJ023 | M | 2 | =907 | LHX-1 | TJ | 2005 Sep | 08-Sep-05 |
| TJ024 | M | 2 | =917 | LHX-1 | TJ | 2006 Feb | 15-Feb-06 |
| TJ026 | M | 2 | =918 | LHX-1 | TJ | 2006 Feb | 16-Feb-06 |
| TJ027 | M | 2 | =919 | LHX-1 | TJ | 2006 Feb | 16-Feb-06 |
| FR039 | F | 2 | =910 | - | FR | 2006 Feb | 18-Feb-06 |
| FR040 | M | 1 | =911 | - | FR | 2006 Feb | 18-Feb-06 |
| FR042 | M | 1 | =912 | - | FR | 2006 Feb | 19-Feb-06 |
| FR043 | M | 1 | =913 | - | FR | 2006 Feb | 19-Feb-06 |
| FR044 | M | 1 | =914 | - | FR | 2006 Feb | 19-Feb-06 |
| FR045 | F | 1 | =915 | - | FR | 2006 Feb | 19-Feb-06 |
| FR047 | F | 1 | =916 | - | FR | 2006 Feb | 21-Feb-06 |
| FR049 | M | 1 | =920 | - | FR | 2006 May | 01-May-06 |
| FR050 | F | 1 | =921 | - | FR | 2006 May | 01-May-06 |
| FR051 | F | 2 | =922 | - | FR | 2006 May | 01-May-06 |
| FR052 | M | 1 | =923 | - | FR | 2006 May | 01-May-06 |
| FR053 | M | 1 | =924 | - | FR | 2006 May | 01-May-06 |
| FR054 | F | 1 | =925 | - | FR | 2006 May | 01-May-06 |
| FR055 | F | 1 | =926 | - | FR | 2006 May | 02-May-06 |
| TJ032 | M | 2 | =936 | LHX-1 | TJ | 2007 Aug | 02-Aug-07 |
| FR056 | M | 3 | =927 | - | FR | 2007 Aug | 03-Aug-07 |
| TJ033 | M | 2 | =935 | LHX-1 | TJ | 2007 Aug | 03-Aug-07 |
| TJ034 | M | 2 | =933 | LHX-1 | TJ | 2007 Aug | 03-Aug-07 |
| TJ035 | M | 2 | =934 | LHX-1 | TJ | 2007 Aug | 03-Aug-07 |
| TJ036 | M | 2 | =932 | LHX-1 | TJ | 2007 Aug | 05-Aug-07 |
| FR057 | M | 1 | =928 | - | FR | 2007 Aug | 08-Aug-07 |
| FR058 | F | 2 | =929 | - | FR | 2007 Aug | 08-Aug-07 |
| FR059 | M | 2 | =930 | - | FR | 2007 Aug | 08-Aug-07 |
| FR060 | M | 2 | =931 | - | FR | 2007 Aug | 08-Aug-07 |
| FR061 | F | 1 | =938 | - | FR | 2007 Aug | 08-Aug-07 |
| TJ038 | M | 2 | =940 | LHX-1 | TJ | 2008 Feb | 25-Feb-08 |
| TJ039 | M | 2 | =941 | LHX-1 | TJ | 2008 Feb | 25-Feb-08 |
| FR062 | M | 1 | =939 | - | FR | 2008 Feb | 27-Feb-08 |
| TJ040 | F | 2 | =942 | LHX-1 | TJ | 2008 Feb | 27-Feb-08 |
| TJ041 | M | 2 | =943 | LHX-1 | TJ | 2008 Feb | 28-Feb-08 |
| TJ043 | F | 2 | =949 | LHX-1 | TJ | 2008 Aug | 22-Aug-08 |
| TJ044 | M | 2 | =944 | LHX-1 | TJ | 2008 Aug | 22-Aug-08 |
| TJ045 | M | 2 | =945 | LHX-1 | TJ | 2008 Aug | 23-Aug-08 |
| TJ046 | M | 2 | =946 | LHX-1 | TJ | 2008 Aug | 23-Aug-08 |
| TJ047 | F | 2 | =947 | LHX-1 | TJ | 2008 Aug | 23-Aug-08 |
| TJ048 | M | 2 | =948 | LHX-1 | TJ | 2008 Aug | 23-Aug-08 |
| TJ050 | M | 1 | =950 | LHX-1 | TJ | 2009 May | 13-May-09 |
| TJ051 | F | 1 | =951 | LHX-1 | TJ | 2009 May | 13-May-09 |
| TJ052 | M | 2 | =952 | LHX-1 | TJ | 2009 May | 13-May-09 |
| TJ053 | M | 1 | =953 | LHX-1 | TJ | 2009 May | 13-May-09 |
| TJ054 | M | 1 | =954 | LHX-1 | TJ | 2009 May | 14-May-09 |
| TJ055 | M | 1 | =955 | LHX-1 | TJ | 2009 May | 14-May-09 |
| TJ056 | M | 2 | =956 | LHX-1 | TJ | 2010 Oct | 20-Oct-10 |
| TJ057 | M | 2 | =957 | LHX-1 | TJ | 2010 Oct | 22-Oct-10 |
| TJ058 | M | 2 | =958 | LHX-1 | TJ | 2010 Oct | 22-Oct-10 |
| TJ059 | M | 2 | =959 | LHX-1 | TJ | 2010 Oct | 23-Oct-10 |
| TJ060 | F | 2 | =960 | LHX-1 | TJ | 2010 Oct | 23-Oct-10 |
| TJ061 | M | 1 | =961 | LHX-1 | TJ | 2011 May | 31-May-11 |
| TJ062 | F | 2 | =962 | LHX-1 | TJ | 2011 May | 31-May-11 |
| TJ063 | M | 1 | =963 | LHX-1 | TJ | 2011 May | 31-May-11 |
| TJ064 | F | 1 | =964 | LHX-1 | TJ | 2011 May | 31-May-11 |
| FR063 | F | 1 | =999 | - | FR | 2011 Jun | 01-Jun-11 |
| FR064 | F | 1 | =998 | - | FR | 2011 Jun | 01-Jun-11 |
| FR065 | F | 1 | =997 | - | FR | 2011 Jun | 01-Jun-11 |

Demographic, brand, cohort, and implantation summary data used to evaluate long-term survival between LHX-1 implanted, temporarily captive (TJ) Steller sea lions and a control of free-ranging juveniles (FR) including sex (Male/Female), age at capture (CapAge), cohort (IndocDate), and group (TJ/FR). This data was used to create covariates for *a priori* Cormack-Jolly-Seber models assessing survival through AICc model selection methods.
