# Supplementary material for: The Effect of Novel Research Activities on Long-term Survival of Temporarily Captive Steller Sea Lions (Eumetopias jubatus)
Source: PLoS One. 2015 Nov 18;10(11):e0141948. doi: 10.1371/journal.pone.0141948 (PMC4651490; doi:10.1371/journal.pone.0141948)
Supplement: S4 Table — Summary of resighting events on an annual basis for both temporarily captive, implanted (prefix TJ-) and free-ranging (prefix FR-) juvenile Steller sea lions. Each individual brand resight history was reduced to a binary encounter history for input into Program MARK for survival analysis. The first instance of a resight or null place holder (-) indicates the year that the animal was marked and released with the resighting events summed in the left column (‘Resights’). Only those resights that included a photograph to confirm a positive identification were included in this analysis. Resights of these study individuals were gathered from various contributing institutions including the National Marine Mammal Laboratory, the Alaska Department of Fish & Game, and the Alaska SeaLife Center. (DOCX) [file pone.0141948.s004.docx]

**S4 Table. Individual brand resighting summary for evaluating survival in juvenile Steller sea lions.**

| **Individual** | **Brand** | **Resights** | **2005** | **2006** | **2007** | **2008** | **2009** | **2010** | **2011** | **2012** | **2013** |
| --- | --- | --- | --- | --- | --- | --- | --- | --- | --- | --- | --- |
| FR035 | =905 | 55 | - | - | 3 | 27 | 2 | 4 | 9 | 3 | 4 |
| FR036 | =904 | 20 | - | 2 | 5 | - | 1 | 3 | 1 | 2 | 4 |
| FR037 | =906 | 18 | - | 5 | 2 | 3 | 1 | 5 | - | - | - |
| FR039 | =910 | 16 |  | 4 | - | - | 3 | - | 1 | 4 | 2 |
| FR040 | =911 | 3 |  | 2 | - | - | - | 1 | - | - | - |
| FR042 | =912 | 12 |  | 7 | - | - | - | 3 | 1 | 1 | - |
| FR043 | =913 | 6 |  | 6 | - | - | - | - | - | - | - |
| FR044 | =914 | 16 |  | 4 | - | 4 | - | 3 | 1 | 2 | 1 |
| FR045 | =915 | 6 |  | 4 | - | 2 | - | - | - | - | - |
| FR047 | =916 | 0 |  | - | - | - | - | - | - | - | - |
| FR049 | =920 | 0 |  | - | - | - | - | - | - | - | - |
| FR050 | =921 | 0 |  | - | - | - | - | - | - | - | - |
| FR051 | =922 | 62 |  | 1 | 3 | 21 | 1 | - | 1 | 34 | 1 |
| FR052 | =923 | 0 |  | - | - | - | - | - | - | - | - |
| FR053 | =924 | 17 |  | - | - | 1 | 1 | 2 | 8 | 3 | 1 |
| FR054 | =925 | 35 |  | 1 | - | 2 | 5 |  | 11 | 6 | 6 |
| FR055 | =926 | 12 |  | 1 | - | - | 1 | 1 | 2 | 2 | 4 |
| FR056 | =927 | 4 |  |  | 1 | 3 | - | - | - | - | - |
| FR057 | =928 | 1 |  |  | 1 | - | - | - | - | - | - |
| FR058 | =929 | 1 |  |  | - | 1 | - | - | - | - | - |
| FR059 | =930 | 13 |  |  | 6 | 7 | - | - | - | - | - |
| FR060 | =931 | 6 |  |  | 2 | 4 | - | - | - | - | - |
| FR061 | =938 | 21 |  |  | - | 3 | 1 | 7 | 3 | 4 | 2 |
| FR062 | =939 | 17 |  |  |  | 6 | 3 | 2 | 3 | 2 | 1 |
| FR063 | =999 | 1 |  |  |  |  |  |  | 1 | - | - |
| FR064 | =998 | 10 |  |  |  |  |  |  | 4 | 3 | 2 |
| FR065 | =997 | 3 |  |  |  |  |  |  | 2 | - | 1 |
| TJ022 | =908 | 24 | - | 2 | - | 5 | 1 | 5 | 2 | 5 | 3 |
| TJ023 | =907 | 0 | - | - | - | - | - | - | - | - | - |
| TJ024 | =917 | 161 |  | - | - | 14 | 5 | 32 | 33 | 25 | 52 |
| TJ026 | =918 | 8 |  | 4 | 1 | - | - | - | 1 | 2 | - |
| TJ027 | =919 | 0 |  | - | - | - | - | - | - | - | - |
| TJ032 | =936 | 15 |  |  | 11 | 4 | - | - | - | - | - |
| TJ033 | =935 | 0 |  |  | - | - | - | - | - | - | - |
| TJ034 | =933 | 63 |  |  | 14 | 35 | 14 | - | - | - | - |
| TJ036 | =932 | 29 |  |  | 8 | 3 | 3 | 3 | 3 | - | 5 |
| TJ038 | =940 | 33 |  |  |  | 8 | 12 | 6 | 1 | 6 | - |
| TJ039 | =941 | 3 |  |  |  | 2 | 1 | - | - | - | - |
| TJ040 | =942 | 344 |  |  |  | 5 | 2 | 6 | 62 | 54 | 214 |
| TJ041 | =943 | 10 |  |  |  | 1 | 1 | 2 | - | 3 | - |
| TJ043 | =949 | 5 |  |  |  | - | 1 | 1 | 3 | - | - |
| TJ044 | =944 | 2 |  |  |  | 1 | 1 | - | - | - | - |
| TJ045 | =945 | 15 |  |  |  | - | 2 | 5 | 3 | 3 | - |
| TJ046 | =946 | 17 |  |  |  | - | 17 | - | - | - | - |
| TJ047 | =947 | 1 |  |  |  | 1 | - | - | - | - | - |
| TJ048 | =948 | 36 |  |  |  | 1 | 3 | 17 | 15 | - | - |
| TJ050 | =950 | 41 |  |  |  |  | 15 | 15 | 3 | 2 | 4 |
| TJ051 | =951 | 32 |  |  |  |  | 32 | - | - | - | - |
| TJ052 | =952 | 58 |  |  |  |  | 37 | 21 | - | - | - |
| TJ053 | =953 | 132 |  |  |  |  | 2 | 17 | 54 | 32 | 25 |
| TJ054 | =954 | 4 |  |  |  |  | 2 | 2 | - | - | - |
| TJ055 | =955 | 47 |  |  |  |  | 2 | 11 | 4 | 17 | 12 |
| TJ056 | =956 | 3 |  |  |  |  |  | - | - | 1 | 2 |
| TJ057 | =957 | 4 |  |  |  |  |  | 1 | 3 | - | - |
| TJ058 | =958 | 2 |  |  |  |  |  | 2 | - | - | - |
| TJ059 | =959 | 25 |  |  |  |  |  | 1 | 23 | 1 | - |
| TJ060 | =960 | 7 |  |  |  |  |  |  | 1 | 4 | 1 |
| TJ061 | =961 | 14 |  |  |  |  |  |  | 4 | 9 | 1 |
| TJ062 | =962 | 3 |  |  |  |  |  |  | 1 | 2 | - |
| TJ063 | =963 | 2 |  |  |  |  |  |  | 2 | - | - |
| TJ064 | =964 | 3 |  |  |  |  |  |  | 3 | - | - |

Summary of resighting events on an annual basis for both temporarily captive, implanted (prefix TJ-) and free-ranging (prefix FR-) juvenile Steller sea lions. Each individual brand resight history was reduced to a binary encounter history for input into Program MARK for survival analysis. The first instance of a resight or null place holder (-) indicates the year that the animal was marked and released with the resighting events summed in the left column (‘Resights’). Only those resights that included a photograph to confirm a positive identification were included in this analysis. Resights of these study individuals were gathered from various contributing institutions including the National Marine Mammal Laboratory, the Alaska Department of Fish & Game, and the Alaska SeaLife Center.
